# Effects of Irradiation During Computed Tomography Scanning on the Function of Implantable Cardioverter-defibrillators

**DOI:** 10.19102/icrm.2024.15073

**Published:** 2024-07-15

**Authors:** Yusaku Nishikawa, Naoki Fujimoto, Tomoaki Kurata, Takashi Sasou, Akio Yamazaki, Yasutaka Ichikawa, Hajime Sakuma, Kaoru Dohi

**Affiliations:** 1 Department of Cardiology and Nephrology, Mie University Graduate School of Medicine, Tsu, Japan; 2 Department of Clinical Engineering, Faculty of Medical Engineering, Suzuka University of Medical Science, Suzuka, Japan; 3 Department of Radiology, Mie University Hospital, Tsu, Japan; 4 Department of Clinical Engineering, Mie University Hospital, Tsu, Japan; 5 Department of Radiology, Mie University Graduate School of Medicine, Tsu, Japan

**Keywords:** Computed tomography scan, electromagnetic interference, implantable cardioverter-defibrillator, inappropriate shock, oversensing

## Abstract

The effect of irradiation during computed tomography (CT) imaging on implantable cardioverter-defibrillators (ICDs) has not been fully evaluated in various settings. The purposes of this study were to evaluate the occurrence of electromagnetic interference (EMI) during CT irradiation in various clinically available ICDs with phantom experiments and to determine the potential risks related to irradiation during CT imaging. Five types of clinically available ICDs from five manufacturers were tested. An ICD was combined with an electrocardiogram (ECG) simulator, mounted in a chest phantom, and subjected to CT imaging. Each ICD was irradiated at the maximal power level (tube voltage, 135 kVp; tube current, 510 mA; rotation time, 1.5 s). EMI was defined as oversensing, ventricular tachycardia/ventricular fibrillation (VT/VF) detection, noise, or shock delivery during CT imaging. For ICDs in which EMI was observed, EMI was then evaluated under 144 different irradiation conditions (tube voltage [four patterns from 80–135 kVp], tube current [six patterns from 50–550 mA], and rotation time [six patterns from 0.35–1.5 s]). Testing was also performed during irradiation at the typical doses in three clinical settings and in two settings with inappropriate irradiation of ICDs due to incorrect setup. Among the five ICDs, a shock was delivered by one ICD manufactured by Medtronic (Minneapolis, MN, USA) due to oversensing during irradiation, which occurred at the maximal power level. No oversensing was observed in other ICDs. In the malfunctioned ICD, oversensing was observed in 134 of 144 irradiation patterns, even at a low power in the ICD. The VF-detection criterion was fulfilled in 20 of 134 tests and was significantly associated with tube voltage, tube current, ration time, and tube voltage × rotation time interaction. Although oversensing was observed in three clinical settings (typical chest CT, CT coronary angiography after coronary artery bypass graft, and dynamic assessment for pleural tumors) and one situation during an incorrect scan range on the chest for head perfusion CT, they were not recognized as tachycardia beats. Oversensing was observed when scans were incorrectly set over the ICD during bolus tracking of contrast-enhanced CT. Maximal power CT imaging induced VT/VF detection and shock delivery in one model of ICD placed in a chest phantom. VT/VF detection was observed when tube voltages were high and irradiation times were longer. Oversensing can occur during inappropriate CT imaging, particularly when slices are positioned over the ICD.

## Introduction

Exposure of pacemakers and implantable cardioverter-defibrillators (ICDs) to X-rays during computed tomography (CT) imaging could cause electromagnetic interference (EMI) phenomena, including oversensing, undersensing, and pace inhibition, and may potentially affect their function. Radiation exposure can also affect the built-in software, resetting the backup mode or changing the programmed parameters.^1,2^ It was reported that oversensing and ventricular fibrillation (VF) detection with charge of the ICD can occur during a CT scan.^3^ Inappropriate shock delivery due to the detection of noise caused by exposure to radiation is considered a significant potential risk in patients with ICDs. To date, no clinical cases of shock occurrence have been reported in observational studies of patients with ICD implantation.^4,5^ However, the number of patients with ICD implantation in the previous studies was <800 at most, and events with a very low incidence (eg, <1 out of 1000) may have been overlooked. An in-depth evaluation by in vitro study is therefore considered necessary to further clarify the potential risks of CT examinations in patients with ICDs and to adequately ensure safety. In a phantom study conducted by McCollough et al. in which ICDs were irradiated at typical clinical doses, oversensing occurred, but no shocks were observed.^2^ However, the models of ICDs used in their study were limited to those made by one manufacturer. Further investigation is needed for the various ICDs that are clinically available.

The purposes of this study were to evaluate the occurrence of EMI during CT imaging in various clinically available ICDs with phantom experiments and to determine potential risks, such as shock delivery, at typical radiation doses used in routine CT imaging protocols.

## Materials and methods

**Figure 1:**
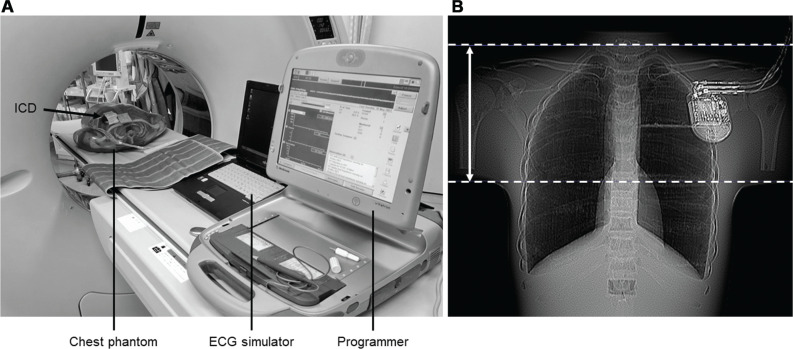
**A:** Experimental setup of the implantable cardioverter-defibrillator (ICD) in the computed tomography room. Each ICD type was combined with an electrocardiogram simulator and mounted individually in the chest phantom. The programmer used the medical implant communication system to communicate with the ICD and print intracardiac electrograms during scanning. **B:** Computed tomography image showing ICD location relative to the internal structures of the phantom. The white arrow shows the irradiation range. *Abbreviations:* ECG, electrocardiogram; ICD, implantable cardioverter-defibrillator.

In this study, the following five different ICD models from five different companies that are clinically available in Japan were examined: the Quadra Assura MP (Abbott, Chicago, IL, USA), Evera XT VR (Medtronic, Minneapolis, MN, USA), Ilivia7 DR-T (Biotronik, Berlin, Germany), Platinium ICD VR 1210 (MicroPort CRM, Clamart, France), and MOMENTUM EL VR D120 (Boston Scientific, Marlborough, MA, USA). Volume CT scanning was performed using an Aquilion ONE 320-detector row scanner (Canon Medical Systems, Ootawara, Japan). The ICD was combined with an electrocardiogram (ECG) simulator (Vip 2; Medtronic) and mounted in a chest phantom (PB-2; Kyoto Kagaku, Kyoto, Japan). The ECG simulator delivered an output of 25 bpm to the ICDs. ICDs were set to the VVI mode at a rate of 30 bpm because pacing at a slow rate shortens the refractory period. Although manufacturer-specific recommended sensitivities for ventricular tachycardia (VT)/VF were reported in the 2015 and 2019 ICD programming guidelines,^6,7^ higher sensitivities for VT/VF were used to detect VT/VF for each model in the present study, as shown in **Table 1**. CT scanning was performed as a volume scan centered on the ICD **(Figures 1A and 1B)**. Using the phantom, a two-stage experiment (experiments 1 and 2) was conducted.

### Definition of oversensing, ventricular tachycardia/ventricular fibrillation detection, and shock delivery during irradiation

**Figure 2:**
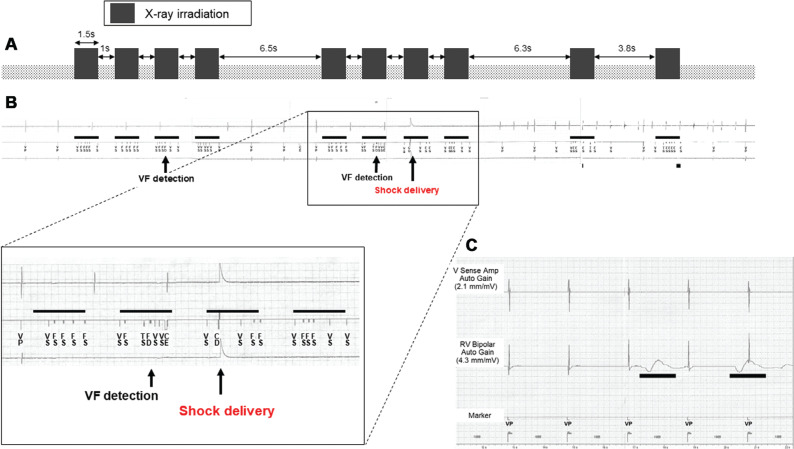
**A:** Diagram of the irradiation schedule. The black bars indicate the times of direct irradiation of the implantable cardioverter-defibrillator, each of which was 1.5 s. To avoid overheating the computed tomography scanner, intervals were inserted between blocks of scans and set to the minimum time. **B:** Electrogram during irradiation shows shock delivery due to oversensing by the Evera XT VR. **C:** Electrogram shows low-frequency noise without oversensing in the Quadra Assura MP. Black bars indicate noise in the right ventricular bipolar waveform. *Abbreviations:* CD, charge delivered; CE, charge end; FD, fibrillation detected; FS, fibrillation-sensed event; RV, right ventricular; TS, tachycardia-sensed event; VF, ventricular fibrillation; VP, ventricular-paced event; VS, ventricular-sensed event.

**Figure 2** shows an electrogram (EGM) recorded during irradiation. Oversensing was defined as the presence of markers other than ventricular-paced events, such as ventricular-sensed events, tachycardia-sensed events, and fibrillation-sensed events, in the EGM. VT/VF detection and shock delivery were recorded as events in the EGM **(Figure 2B)**. EMI was defined as oversensing, VT/VF detection, and shock delivery during CT imaging.

In experiment 1, the effects of direct irradiation at a maximum power level (tube voltage, 135 kVp; tube current, 510 mA; rotation time, 1.5 s) of the CT system on the ICDs were evaluated. As shown in **Figures 2A and 2B**, each ICD was irradiated 10 times (1.5 s × 10) at the maximal power, and the occurrence of EMI during the CT scans was evaluated. The time intervals between scans to avoid overheating of the CT scanner were set as the minimum values **(Figure 2A)**.

**Figure 3:**
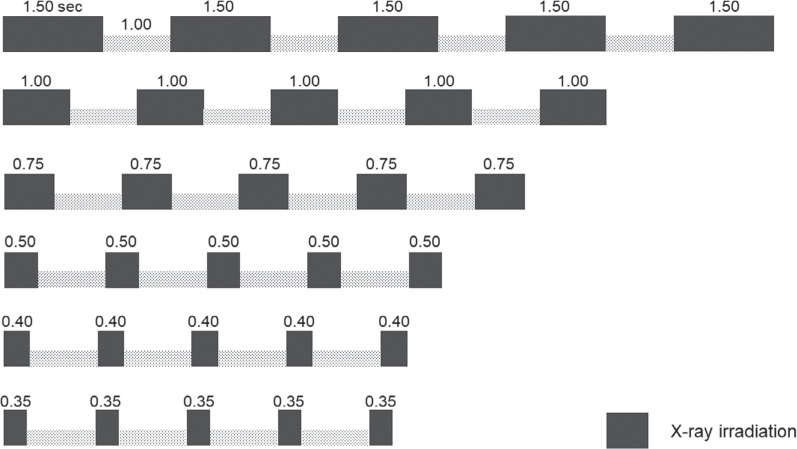
Six patterns of rotation time settings in experiment 2. Black bars indicate irradiation time (s); five irradiations were repeated regularly. The interval between each irradiation was 1 s for all patterns.

**Figure 4:**
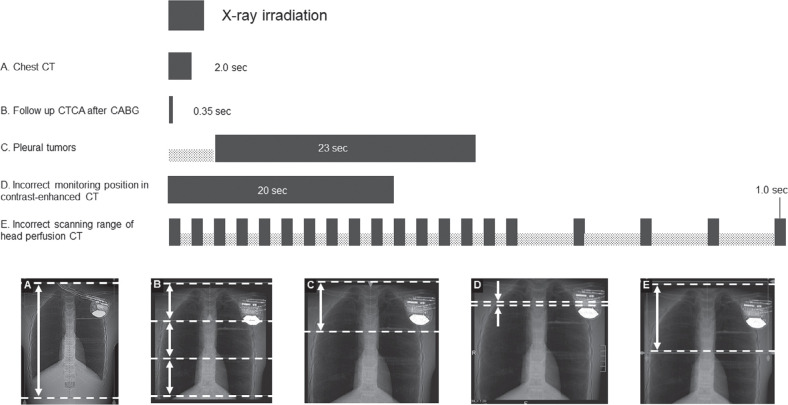
Time of direct irradiation on the implantable cardioverter-defibrillator at each of three clinical settings **(A–C)** and two inappropriate scanning settings **(D, E)**. The computed tomography images at the bottom show the ICD location relative to the internal structures of the phantom for each setting. The white arrow shows the irradiation range. In **B**, the three white arrows indicate the scan areas within the volume scan. In **D**, slices were set directly over the integrated circuit of the ICD. *Abbreviations:* CABG, coronary artery bypass graft; CT, computed tomography; CTCA, computed tomography coronary angiography; ICD, implantable cardioverter-defibrillator.

In experiment 2, ICDs that exhibited EMI in experiment 1 were irradiated according to various CT imaging parameters and assessed for the occurrence of EMI during each CT scan. A total of 149 patterns of CT imaging parameters were used for the experiment, including the 144 patterns shown in **Table 2** and **Figure 3** (tube voltage [four patterns from 80–135 kVp], tube current [six patterns from 50–550 mA], and rotation time [six patterns from 0.35–1.5 s]) and the 5 patterns for routine CT protocols shown in **Table 3** and **Figure 4**: the irradiation duration per one scan for typical chest CT; follow-up coronary CT angiography after coronary artery bypass graft; dynamic assessment for pleural tumors; the monitoring position set incorrectly for the bolus-tracking technique for contrast-enhanced CT; and the incorrect scan range for head perfusion CT of 2.0, 0.35, 23, 20, and 1.0 s, respectively **(Figure 4)**. ICDs were irradiated 5 times in the 144 patterns of CT imaging parameters and 10 times in the 5 patterns for routine CT protocols, respectively.

All CT scans were performed by two radiologic technologists (A.Y. and T.K.). The manipulation of the ICDs and the analysis of EMI during the CT scans were performed by one of the authors (Y.N.). None of the authors have conflicts of interest with Abbott, Medtronic, Biotronik, Micro Port, or Boston Scientific.

### Statistical analysis

Data analyses were performed using SPSS 28.0 (IBM Corp., Armonk, NY, USA). A three-way analysis of variance (ANOVA) with post hoc test was used to evaluate the effects of three factors (tube voltage, tube current, and rotation time) on the occurrence of oversensing and VT/VF documentation, respectively. Statistical significance was set at *P* < .05.

## Results

### Experiment 1

At the maximum power level of the CT system, EMI was observed in one of the five ICDs (Evera XT VR; Medtronic). The EMI was a shock delivery due to oversensing in the tip-to-coil configuration **(Figures 2A and 2B)**. Shock delivery by the ICD occurred during successive scans as a result of the addition of the oversensing signal being misrecognized as VF, but not during a single exposure for 1.5 s **(Figure 2B)**. No shock delivery was observed in the tip-to-ring configuration; however, oversensing was observed in this context. Although low-frequency fluctuation of the signal was observed in the Quadra Assura MP (Abbott), it was not recognized as ventricular beats or another kind of EMI **(Figure 2C)**. No EMI was observed in any other ICD model.

### Experiment 2

**Figure 5:**
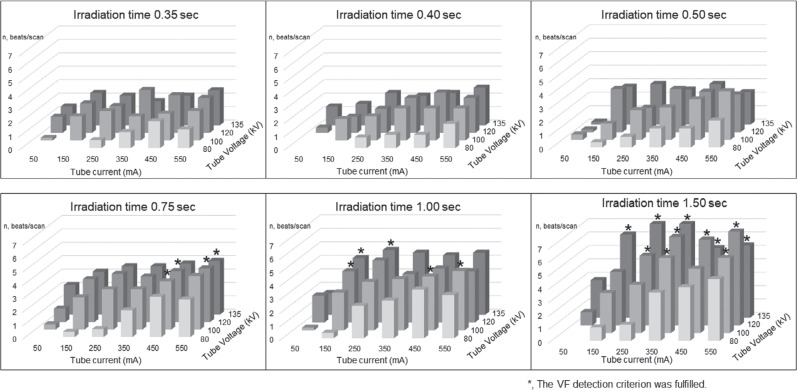
Electromagnetic interference according to settings for the parameters of exposure time, tube current, and tube voltage. The vertical axis is the number of “tip-to-coil” oversensing events, converted to the number of times per scan. ^a^Ventricular tachycardia/fibrillation or oversensing detected as noise by the implantable cardioverter-defibrillator.

In experiment 2, one ICD model (Evera XT VR), which exhibited EMI in experiment 1, was examined. **Figure 5** shows the number of oversensing events that occurred during each of the 144 patterns of irradiation for this ICD model. Of the 144 patterns of irradiation, “tip-to-ring” oversensing was observed in 140 patterns (97.2%) and “tip-to-coil” oversensing was observed in 134 patterns (93.1%). Three-way ANOVA showed significant effects on the oversensing for three factors: tube voltage, tube current, and rotation time (all *P* < .001). Significant interaction effects were also observed between the following factor pairs in the occurrence of oversensing: tube voltage × rotation time, tube voltage × tube current, and tube current × rotation time (*P* < .001).

The number of patterns in which oversensed beats fulfilled the VT/VF detection criteria was 9 out of 140 (6.4%) for the “tip-to-ring” method and 20 out of 134 (14.9%) for the “tip-to-coil” method. **Figure 5** shows the number of “tip-to-coil” oversensing events, converted to the number of times per scan. VT/VF due to oversensing was not detected at irradiation durations of ≤0.5 s. A three-way ANOVA showed significant effects for three factors: tube voltage, tube current, and rotation time (all *P* ≤ .014). A significant interaction effect in VT/VF detection was observed between the factors of tube voltage × rotation time (*P* = .001), while no significant interaction was found between tube voltage × tube current (*P* = .80) or tube current × rotation time (*P* = .06).

Oversensed beats were observed during typical chest CT (3.2 beats/scan), follow-up coronary CT angiography after coronary artery bypass graft (4.8 beats/scan), and dynamic assessment for pleural tumors (19.2 beats/scan). However, no VT/VF or fluctuation of signals was detected in these settings. An average of 35.2 oversensed beats per scan was observed when the scan range was incorrectly set at the ICD during the 20 s of bolus tracking for contrast-enhanced CT. However, these signals were recognized by the ICD as noise but not VT/VF. Although oversensing was observed in the incorrect scan range for head perfusion CT (14.2 beats/scan), it was not detected as VT/VF.

## Discussion

The occurrence of EMI during CT irradiation in a variety of clinically available ICDs was investigated in this study. The phantom experiment demonstrated that CT scanning under maximum radiation dose conditions caused VT/VF detection in one commercially available ICD. The VT/VF detection was significantly associated with higher tube voltage and longer irradiation time. For the occurrence of oversensing in the ICD, there was a significant relationship with higher tube currents and tube voltages. The number of oversensing events significantly increased with an increase in the irradiation time of the ICD. Another important finding was that oversensing can also occur if the ICD is scanned incorrectly under the CT scanning conditions used in clinical practice.

### Computed tomography-induced electromagnetic interference among manufacturers

We observed oversensing in the Evera XT VR model (Medtronic) during maximal power CT scanning but not in the other four models. This may be due to differences in amplifiers when processing the input waveforms, each of which have their own algorithm. In fact, in the Quadra Assura MP model (Abbott), low-frequency signal fluctuations were observed in the right ventricular bipolar EGM but not in the V Sense Amp EGM **(Figure 2C)**. The right ventricular bipolar potential was measured on the EGM prior to passing through the amplifier. In contrast, the V Sense Amp EGM was measured after passing through the amplifier. We speculate that, during the measurements of the V Sense Amp EGM, any signal fluctuation was removed by the amplifier.

Several phantom studies reported oversensing in pacemakers and ICDs manufactured by Medtronic.^1,2^ McCollough et al. previously evaluated EMI in eight ICDs under both maximum and typical dose levels of CT irradiation using a phantom. They reported that maximum-dose irradiation caused serious events such as ventricular safety pacing and partial electrical reset in some models, whereas typical-dose irradiation caused oversensing for only several seconds.^2^ These results appeared to be consistent with our results. Tsutsui et al. reported using data from 1259 patients with pacemakers and ICDs from several companies that irradiation during CT scans induced oversensing and/or reset in 19 of 2362 scans (18 oversensing and/or reset in pacemakers and 1 oversensing in an ICD).^5^ All these EMIs were observed in pacemakers or ICDs manufactured by Medtronic. The observed EMIs in pacemakers included device reset in the Insync 8040 (14 scans) and oversensing in the Kappa (4 scans). Oversensing without a pre-shock charge was observed in the InSync III Marquis,^5^ which is an older type of ICD compared to those used in the present study. The ICDs reported in these previous studies were older models, which may partly explain why EMIs were induced by CT irradiation in the clinical setting. As new models of ICDs are introduced into clinical practice, the effects of irradiation on ICDs should be updated in an appropriate manner.

### Effects of computed tomography irradiation on shock delivery

In experiment 1, we observed VT detection and shock delivery at the maximal level of CT scanning. Although CT-induced inappropriate shock delivery is considered a significant potential risk in patients with ICDs, it has not been fully studied. For example, the current expert consensus referenced only one clinical study in which the impacts of CT scanning on inappropriate shocks were evaluated in patients with ICDs.^8^ There was another clinical study reported after said expert consensus. In these two studies (one with 241 patients by Hussein et al. and the other with 536 patients by Tsutsui et al.), inappropriate shock delivery was not observed.^4,5^ In contrast, Porres et al. reported a case with a pre-shock ICD charge due to CT-induced oversensing.^3^ The total number of patients with ICDs enrolled in the previous two studies was <800. Therefore, a very low incidence of the event could not have been overlooked.

### Effects of tube current, tube voltage, and irradiation time on oversensing and ventricular tachycardia/ventricular fibrillation detection

We observed that high tube voltage and tube current induced oversensing and that the number of oversensing events increased with longer irradiation times, respectively. We also observed that the tube voltage × rotation time interaction was significantly associated with the VT/VF documentation. In an experiment using a Medtronic model pacemaker, Umezawa et al. reported that X-ray–induced EMI was dependent on tube voltage, tube current, and X-ray dose.^9^ In the present experiment 2, low-power CT scans showed slight oversensing but no VT/VF detection **(Figure 5)**. Consistent with our results regarding irradiation time, expert consensus suggests that attention should be paid to scans with longer irradiation times, such as four-dimensional CT and cone-beam CT.^8^ Although a longer irradiation time (23 s) was needed for dynamic assessment of pleural tumors, the tube voltage (80 kVp) and tube current (100 mA) were relatively low, and no VT/VF was detected **(Table 3, Figure 4C)**.

The ICD needs time to recognize VT requiring shock and then to deliver the electric shock. In patients with an ICD or cardiac resynchronization therapy defibrillator, reducing the number of shocks has been reported to be associated with improved mortality and quality of life.^10,11^ In addition, extending the detection time may reduce the number of ICD shocks and improve survival.^12–14^ An optimal ICD program has been published by each manufacturer.^7^ In our daily clinic, these evidence-based ICD settings and shortened irradiation time may reduce the risk of inappropriate shocks during CT scanning.

### Prolonged irradiation of implantable cardioverter-defibrillators in the clinical setting

We observed oversensing when the monitoring position was set incorrectly for the bolus-tracking technique for contrast-enhanced CT, as has been described previously. In this setting, as shown in **Figure 4D**, a series of dynamic low-dose monitoring scans had been improperly acquired over the ICD. The present result indicates that the risk of irradiation to the ICD needs to be considered, even if the main purpose of the CT examination is a scan that does not irradiate the ICD. Other studies have reported prolonged CT examinations due to human error,^15^ as well as complications such as hair loss and skin erythema that were caused by CT scans with the proper settings.^16,17^ Careful attention should be paid to patients with ICDs other than pacemakers because of the risks of EMI caused by irradiation if the scan range is set incorrectly.

### Clinical implications

The present findings suggest that unnecessary shock is rarely delivered by currently available ICDs in CT examinations that have a short duration of irradiation. However, depending on the ICD model, radiation exposure could result in oversensing or false detection of VT/VF. Oversensing is associated with higher tube current, higher tube voltages, and longer irradiation durations, while misidentification of VT/VF is associated with higher tube voltages and longer irradiation durations. Oversensing can occur even in clinically used CT protocols due to improper imaging coverage that includes the ICD in the scan area. The present study reveals potential risks from irradiation in a variety of ICDs available in routine clinical practice.

In Japan, deactivation of defibrillation functions is mandatory not only during magnetic resonance imaging but also CT examinations if the ICD is directly irradiated. However, our results could suggest that deactivation is unnecessary in daily practice, although prolonged direct irradiation of ICDs should be avoided as much as possible, as in Europe and the United States. We believe that deactivation of defibrillation functions will be discussed in Japan, especially as the present results may have a great impact on clinical practice in Japan.

### Limitations

There are some limitations to the present study. We evaluated one ICD from each of five manufacturers. Different models may employ different integrated circuit technologies in the design of the product, and the incidence of CT-induced EMI may also differ among the ICD designs. Thus, we cannot rule out EMIs in ICDs from manufacturers other than Medtronic. As we chose maximum levels of irradiation and higher sensitivities for VT/VF, the effects observed in the present study might be due to the experimental setup with a special clinical situation. The ICDs used in this study are no longer the newest ICDs currently on the market. Newer models of ICDs are commercially available, and even newer models will be introduced in the future. These ICDs may have better EMI protection than those used in the present study. In addition, each experiment was done once on each ICD device. Therefore, reproducibility of EMI due to irradiation was not evaluated in the present study.

## Conclusions

CT scanning at the maximal power induced VT/VF in an ICD. VT/VF was detected more frequently in settings of higher tube voltages and longer times of direct irradiation to the ICD. Oversensing can occur during inappropriate CT scanning, especially if scans are positioned over the ICD.

## Figures and Tables

**Table 1:** Implantable Cardioverter-defibrillator Programming

	Quadra Assura MP	Evera XT VR	Platinium ICD VR 1210	Ilivia7 DR-T	MOMENTUM EL VR D120
Zone	VT/VF	VT/VF	Slow VT/VT/VF	VT/VF	VT/VF
Rate (per min)	101/139	92/150	100/130/150	100/150	90/110
VT detection (intervals)	8	12	4/4	10	6/8 (1 s)^a^
VF detection (intervals)	12	12/16	4	6/8	6/8 (1 s)^a^
ATP	None	None	None	None	None
Shock energy (J)	0.1	0.4	0.5	2	0.1
Basic interval (per min)	30	30	30	30	30
Pacing pulse amplitude (V)	2.5	2.5	2.5	2.5	2.5
Pacing pulse width (ms)	0.5	0.4	0.35	0.4	0.35
Sensing polarity	Fixed	Tip to ring, tip to coil	Fixed	Fixed	Fixed

*Abbreviations:* ATP, anti-tachycardia pacing; ICD, implantable cardioverter-defibrillator; VF, ventricular fibrillation; VT, ventricular tachycardia. ^a^The interval for detection is fixed, and the delay time is set. Shock delivery requires that this time rhythm must be sustained after detection.

**Table 2:** Computed Tomography Scan Parameters

Tube Voltage (kVp)	Tube Current (mA)	Rotation Time (s)
80	50	0.35
100	150	0.40
120	250	0.50
135	350	0.75
	450	1.00
	550 (510)^a^	1.50

^a^The upper limit of the tube current is 510 mA when the tube voltage is 135 kVp.

**Table 3:** Parameters Used in Three Clinical Settings and in Two Inappropriate Computed Tomography Scans

Situation	Tube Voltage, kVp	Tube Current, mA	Rotation Time, s	Maximum Continuous Irradiation Time, s	Scan Method
Chest CT	120	150	0.50	2.0	Helical scan
Follow-up CTCA after CABG	120	550	0.35	0.35	Volume scan
Dynamic assessment for pleural tumors	80	100	0.35	23	Volume scan
Incorrect monitoring position in contrast-enhanced CT	120	50	0.5	20	Confirmation before CT scan
Incorrect scanning range of head perfusion CT	80	100	1.0	1.0	Volume scan

*Abbreviations:* CABG, coronary artery bypass grafting; CT, computed tomography; CTCA, computed tomography coronary angiography.
